# Using Survey Data for Diabetes Surveillance Among Minority Populations: A Report of the Centers for Disease Control and Prevention’s Expert Panel Meeting

**Published:** 2004-03-15

**Authors:** Nilka Ríos Burrows, José Lojo, Michael M. Engelgau, Linda S. Geiss

**Affiliations:** Division of Diabetes Translation, National Center for Chronic Disease Prevention and Health Promotion, Centers for Disease Control and Prevention

## Abstract

**Introduction:**

Data on diabetes morbidity and mortality and the quality of care among U.S. minority populations are necessary to assess progress toward eliminating racial/ethnic disparities and to design and implement effective interventions. This paper summarizes the discussions and recommendations of an expert panel to address the use of survey data for diabetes surveillance among minority populations.

**Methods:**

The Centers for Disease Control and Prevention's Division of Diabetes Translation convened an expert panel of persons with survey experience and awareness of the problems in conducting health-related surveys among minority populations. Panel members were asked to 1) determine ways to enhance the ability of existing survey systems to address diabetes surveillance among minority populations; 2) identify survey systems that could be used to address surveillance needs; and 3) determine whether new minority-specific survey systems need to be developed.

**Results:**

Panel members concluded that, although no existing survey system is completely adequate for diabetes surveillance among minority populations, new systems should not be developed. They recommended 1) investigating the use of community-based surveys; 2) exploring the ability of national surveys to increase sample sizes and produce state-level estimates; and 3) encouraging government agencies and public health programs to coordinate and integrate diabetes-related survey data and share analytic methodology.

**Conclusion:**

No existing survey is suitable for conducting minority-specific diabetes surveillance. Modifying and expanding existing surveys to establish a diabetes surveillance system of sentinel minority populations would be more feasible than developing a new one. Interagency coordination and collaboration will be critical in this effort.

## Introduction

An estimated 17 million people in this country have diabetes, and of these, nearly 6 million are not aware of their condition (1). According to the American Diabetes Association, diabetes costs nearly $100 billion in direct medical care costs and indirect costs such as lost productivity (2). Appropriate preventive care practices could prevent or delay a large proportion of the costly and disabling consequences of diabetes (3).

Compared with the general population, certain racial/ethnic communities, as well as older Americans and economically disadvantaged Americans, are disproportionately affected by diabetes and are at increased risk for some diabetes-related complications (4). Potential reasons for these groups being disproportionately affected include a greater prevalence of risk factors and comorbid conditions, inadequate access to medical care, and suboptimal diabetes-related preventive care.

The Division of Diabetes Translation (DDT) at the Centers for Disease Control and Prevention (CDC) uses many data sources to conduct public health surveillance of diabetes and to estimate the burden of diabetes at the national and state levels. These data sources include surveys such as the Behavioral Risk Factor Surveillance System (BRFSS) and the National Health Interview Survey (NHIS), administrative databases such as the U.S. Renal Data System, databases of the Health Care Financing Administration, and vital statistics data. However, several problems are associated with the use of these data in diabetes surveillance among minority populations: 1) surveys cannot reach all minority populations of interest; 2) administrative data sometimes do not include information on race and ethnicity; 3) small sample sizes of minority populations do not allow for accurate estimates of the diabetes burden at the state or community level; and 4) minority populations often are erroneously treated as homogeneous (for example, Mexican Americans, Cuban Americans, and other distinct ethnic groups are usually considered to be part of a homogenous group called "Hispanics").

In 1998, the Department of Health and Human Services' Initiative to Eliminate Racial and Ethnic Disparities in Health established as a national priority the elimination of racial and ethnic disparities in health outcomes among U.S. residents. Data for monitoring progress toward this objective, however, are lacking for many minority populations. Obtaining high-quality surveillance data on diabetes-related morbidity and mortality and quality of diabetes care among minority populations is thus important in identifying disparities and monitoring progress toward reducing these disparities. To discuss the use of survey data for the surveillance of diabetes among minority populations, DDT convened an expert panel meeting.

## Methods

DDT organized and conducted the August 5–6, 2002, expert panel meeting. The panel consisted of 18 persons from agencies or organizations outside of DDT, most of whom had survey experience and were aware of the challenges in conducting surveys of health-related information among minority populations. Panel members represented 4 federal agencies, 4 university-affiliated institutions, 2 private nonprofit organizations, and one state health department (Table 1). Panel members were asked to help the CDC to 1) determine ways to enhance the ability of existing survey systems to address diabetes surveillance among minority populations; 2) identify survey systems that could be used, but currently were not being used, to address surveillance needs; and 3) determine whether new minority-specific survey systems need to be developed.

## Results

The expert panel members identified numerous national, state, and community-based survey systems and discussed the systems' abilities to address the health information needs of minority populations (Table 2). Most of the identified surveys were cross-sectional in design and national in scope, administered either face-to-face or by telephone, and sponsored or supported by various federal agencies. The expert panel members noted several issues and problems associated with using national survey data for disease and risk factor surveillance. These included sample sizes for minority populations being too small, sampling being limited to the larger minority populations, discrete minority groups being treated as homogeneous rather than diverse populations, and national data being inadequate for estimating state and community problems.

Moreover, the panel questioned whether a national sample of specific minority populations could produce meaningful results given the diversity within these populations. State and community surveys share some of these same problems, but they have the advantage of access to local data, which have more immediate relevancy in planning and evaluating community-based interventions to improve public health. Although local surveys may have the greatest potential for targeting subgroups of minorities, data on such subgroups may not be generalizable to the larger minority populations.

The panel members recognized that the list of surveys discussed as potential sources of data was probably not complete and recommended several strategies to identify additional survey systems that could be useful. These strategies included 1) a summary review of existing longitudinal studies and other relevant data; 2) a search of surveys sponsored by federal or public agencies other than the Department of Health and Human Services; and 3) a query of state health programs to establish an inventory of special surveys.

Recognizing that diabetes is an important growing public health problem (5), the panel stressed that the inclusion of diabetes-related data in existing survey systems is critical. The panel highlighted the need to coordinate the diabetes surveillance efforts of survey systems by using uniform diabetes-related questions, sharing analytical techniques (such as pooling data), and promoting the standardization of measurement and analysis practices.

The panel also discussed the need to examine survey content to ensure that surveys are capable of producing the thorough data necessary to design effective public health interventions. In addition to producing data on minority racial and ethnic groups, surveys also should produce data on other disadvantaged populations as measured by socioeconomic status, social capital, community resources, education, and access to or denial of health insurance, because these factors generally underlie many racial and ethnic disparities.

Finally, the panel discussed the need to establish new minority-specific survey systems and concluded that developing and maintaining new survey systems would be too costly and time-consuming. Instead, the panel recommended expanding upon and enhancing existing survey systems at the state and local levels. Specifically, the panel recommended investigating the use of community-based surveys, such as those in the Racial and Ethnic Approaches to Community Health 2010 project, state- and local-specific surveys of the BRFSS, and the State and Local Area Integrated Telephone Survey. The panel also suggested modifying existing surveys by, for example, increasing sample size, adding supplementary content, sampling additional minority groups, and developing the capacity of national surveys such as NHIS to collect state and community-level data.

## Discussion

Data on diabetes-related morbidity and mortality and quality of diabetes care among different U.S. minority populations are necessary to 1) assess progress toward eliminating racial/ethnic disparities in the health burden of diabetes, and 2) design and implement effective interventions for minority groups that are disproportionately affected by diabetes.

No existing survey is suitable for conducting minority-specific diabetes surveillance. Modifying and expanding existing surveys to establish a diabetes surveillance system of sentinel minority populations, however, would be more feasible than developing a new one. Interagency coordination and collaboration will be critical in establishing such a system.

## Figures and Tables

**Table 1:** Members of the Centers for Disease Control and Prevention's Expert Panel on Using Survey Data for Diabetes Surveillance Among Minority Populations, 2002

**Centers for Disease Control and Prevention**	**Other Federal Agencies**	**University-affiliated Institution**	**Private Nonprofit Organization**	**State Health Department**
Lawrence Barker, PhD Stephen J. Blumberg, PhD Nilka Ríos Burrows, MPH Michael M. Engelgau, MD, MS Clark Denny, PhD Linda S. Geiss, MA H. Wayne Giles, MD Howard Goldberg, PhD José Lojo, MPH Ali H. Mokdad, PhD Kathryn S. Porter, MD, MS Edward F. Tierney, MPH Elizabeth Zell, MStat	Karen Beauregard, MHA, Agency for Healthcare Research and Quality	Charlotte Steeh, PhD, Georgia State University, School of Policy Studies	Heather DH Mann, MA, National Indian Council on Aging	Robert W. Indian, MS, Ohio Department of Health
	Gerald S. Adler, MPhil, Centers for Medicare And Medicaid Services	Carol-Ann Emmons, PhD University of Chicago, National Opinion Research Center	Robert Valdez, PhD, Rand Institute	
	Adrienne Oneto, MA, U.S. Census Bureau	David Weir, PhD, University of Michigan, Institute for Social Research		
		Elisa T. Lee, PhD, University of Oklahoma, Health Sciences Center		

**Table 2:** Description of National, State and Community-based Survey Systems Relevant to Addressing Health Information Needs of Minority Populations, Centers forDisease Control and Prevention, 2002

**National Surveys**	**Agency^a^**	**Target Population**	**Survey Design**	**Survey Mode**	**Frequency**
American Community Survey	U.S. Census Bureau	Civilian, non-institutionalized household population	Cross-sectional	Mail, phone, person	Annual beginning in 2003
Health and Retirement Study	NIA	Civilian, non-institutionalized household population aged >50 years	Panel	Person	Every 2 years
Medicare Current Beneficiary Survey (MCBS)	CMS	Medicare beneficiaries aged >64 years	Panel	Person (computer-assisted)	3 times/year for 4 years
Medical Expenditure Panel Survey (MEPS)	AHRQ	Civilian, non-institutionalized household population and nursing home residents	Panel	Person (computer-assisted)	Several times/year for 2 years
National Survey Family Growth	CDC	Civilian, non-institutionalized population aged 15-44 years	Cross-sectional	Person	Periodic
National Health Interview Survey (NHIS)	CDC	Civilian, non-institutionalized household population	Cross-sectional	Person	Annually
National Health and Nutrition Examination Survey (NHANES)	CDC	Civilian, non-institutionalized household population	Cross-sectional	Person	Periodic
Consumer Assessment of Health Plans Survey (CAHPS)	AHRQ	Persons currently enrolled in health plans	Cross-sectional	Phone (computer-assisted)	To be determined

**State and Community-based Surveys**	**Agency**	**Target Population**	**Survey Design**	**Survey Mode**	**Frequency**
State and Local Area Integrated Telephone Survey (SLAITS)	CDC	Subgroups of civilian, non-institutionalized household population (e.g., low-income households)	Cross-sectional	Phone	Annual
Behavioral Risk Factor Surveillance System (BRFSS)	CDC	State-based, civilian, non-institutionalized household population aged >17 years	Cross-sectional	Phone	Annual
Youth Risk Behavior Survey	CDC	Students in grades 9-12	Cross-sectional	Person	Every 2 years
BRFSS special surveys	State	Civilian, non-institutionalized population aged >17 years	Cross-sectional	As specified by state	As specified by state
Racial/Ethnic Approaches to Community Health (REACH 2010)	CDC	21 minority communities per the REACH 2010 grantees	Cross-sectional	Phone	Annually for 4 years

a AHRQ: Agency for Healthcare Research and Quality; CDC: Centers for Disease Control and Prevention; CMS: Centers for Medicare and Medicaid Services; NIA: National Institute on Aging.
